# Topochemical conversion of an imine- into a thiazole-linked covalent organic framework enabling real structure analysis

**DOI:** 10.1038/s41467-018-04979-y

**Published:** 2018-07-03

**Authors:** Frederik Haase, Erik Troschke, Gökcen Savasci, Tanmay Banerjee, Viola Duppel, Susanne Dörfler, Martin M. J. Grundei, Asbjörn M. Burow, Christian Ochsenfeld, Stefan Kaskel, Bettina V. Lotsch

**Affiliations:** 1 Max Planck Institute for Solid State Research, Heisenbergstraße 1, 70569 Stuttgart, Germany; 2 Department of Chemistry, Ludwig-Maximilians-Universität München, Butenandtstr. 5-13, 81377 Munich, Germany; 3 Department of Inorganic Chemistry 1, TU Dresden, Bergstraße 66, 01069 Dresden, Germany; 4 Fraunhofer Institute for Material and Beam Technology (IWS), Winterbergstr. 28, 01277 Dresden, Germany

## Abstract

Stabilization of covalent organic frameworks (COFs) by post-synthetic locking strategies is a powerful tool to push the limits of COF utilization, which are imposed by the reversible COF linkage. Here we introduce a sulfur-assisted chemical conversion of a two-dimensional imine-linked COF into a thiazole-linked COF, with full retention of crystallinity and porosity. This post-synthetic modification entails significantly enhanced chemical and electron beam stability, enabling investigation of the real framework structure at a high level of detail. An in-depth study by electron diffraction and transmission electron microscopy reveals a myriad of previously unknown or unverified structural features such as grain boundaries and edge dislocations, which are likely generic to the in-plane structure of 2D COFs. The visualization of such real structural features is key to understand, design and control structure–property relationships in COFs, which can have major implications for adsorption, catalytic, and transport properties of such crystalline porous polymers.

## Abstract (web summary)

Stabilization of covalent organic frameworks (COFs) by post-synthetic locking is a powerful tool to push the limits of COF utilization. Here the authors demonstrate a sulfur-assisted conversion of an imine-linked COF into a thiazole-linked COF, with retention of crystallinity and porosity, allowing for direct imaging of defects in COFs.

## Introduction

Covalent organic frameworks (COFs) distinguish themselves from conventional polymers by three defining features: covalent connectivity, porosity, and crystallinity. This generation of two- (2D) and three-dimensional (3D) polymers is synthesized under solvothermal conditions by reversible covalent bond forming reactions^1–3^. Reversibility of the COF linkage is key to obtain ordered materials by error correction and defect healing^4–6^, but at the same time makes COFs inherently unstable and rich in defects. This dilemma can be circumvented by performing a reversible order-inducing step under thermodynamic control and subsequently arresting this order via a post-synthetic treatment, a concept that has been explored for the synthesis of crystalline, “unfeasible” zeolites^7^ as well as to stabilize the molecular cages formed by dynamic covalent chemistry^8^. In order to arrest COFs in their crystalline state (arrested linkage COFs: ALCOFs) the chemical linkage of the COF needs to be converted^2^ from a reversible to an irreversible type of bond in a topochemical fashion.

The competition between reversibility and stability, which is dominated by subtle changes in the reaction conditions, often leads to COFs with only moderate chemical stability and low crystallinity. As a result, nanocrystalline COFs with ubiquitous structural disorder are obtained. Such structural disorder is difficult to probe or even quantify by conventional diffraction methods such as X-ray powder diffraction (XRPD), while imaging with transmission electron microscopy (TEM) is difficult due to the sensitivity of COFs – similar to most soft matter systems – to the electron beam in comparison to inorganic crystalline materials^1,4,9^. In spite of these challenges, analysis of the ideal and real structure of COFs has been in the focus since the very beginning of the field, and it continues to attract attention as the field^1,4,9–12^.

In 2D COFs, various kinds of out-of-plane disorder are prevalent, such as random or poorly defined stacking sequences^4,13,14^. In-plane defects, which also impact the electronic transport properties of COFs, have been invoked to explain the low crystallinity of many COFs^15^. In closely related systems such as on-surface grown, monolayer 2D polymers, rings with five or seven edges in an otherwise hexagonal system have been observed as defects by scanning tunneling microscopy^16,17^, very similar to the formation of defects in graphene^18^. While often detrimental, such defects can be highly beneficial, for example in (electro)catalysis where they act as high-energy binding sites for the adsorption of reaction intermediates^19^. In addition, the typically limited size of the crystallites of COFs leads to grain boundaries and domain intergrowth, which can drastically influence the long-range charge carrier percolation in COFs^20^ as well as influence ion conduction^21,22^. These examples show that disorder can have a significant influence on the chemical, structural and (opto-)electronic properties of COFs. However, such defects were never directly imaged. Hence, it is of paramount importance to understand the types of defects and disorder present in COFs and to elucidate and control their influence on the properties of the material.

In this work, we investigate the topochemical conversion of the triphenyl triazine imine COF (TTI-COF)^4,23^ with elemental sulfur into a thiazole-linked COF through a post-synthetic locking strategy, thereby establishing a class of thiazole-based COFs. This type of post-synthetic modification is fundamentally different from previous examples, such as the introduction of functionalities by tethering side groups^24^ or heterogeneous linker exchange:^25^ These approaches either do not change the reversible bond of the starting COFs, or they even utilize the reversibility to introduce functionality. The post-synthetic oxidation of an imine COF linkage to an amide, as reported recently, provides a direct transformation of the reversible bond of the COF;^2^ however it was recently shown that even amides are, in principle, reversible enough to be used for the synthesis of COFs^26^. Modification of the imine linkage of the TTI-COF leads to excellent contrast and high electron beam stability of the sulfur-modified TTI-COF (triphenyl triazine thiazole COF; TTT-COF), which enables imaging and analysis of in-plane defects with TEM, thus revealing details of real structure effects that have not been amenable to direct observation in any COF so far.

## Results

### Post-synthetic locking by imine to thiazole conversion

**Fig. 1:**
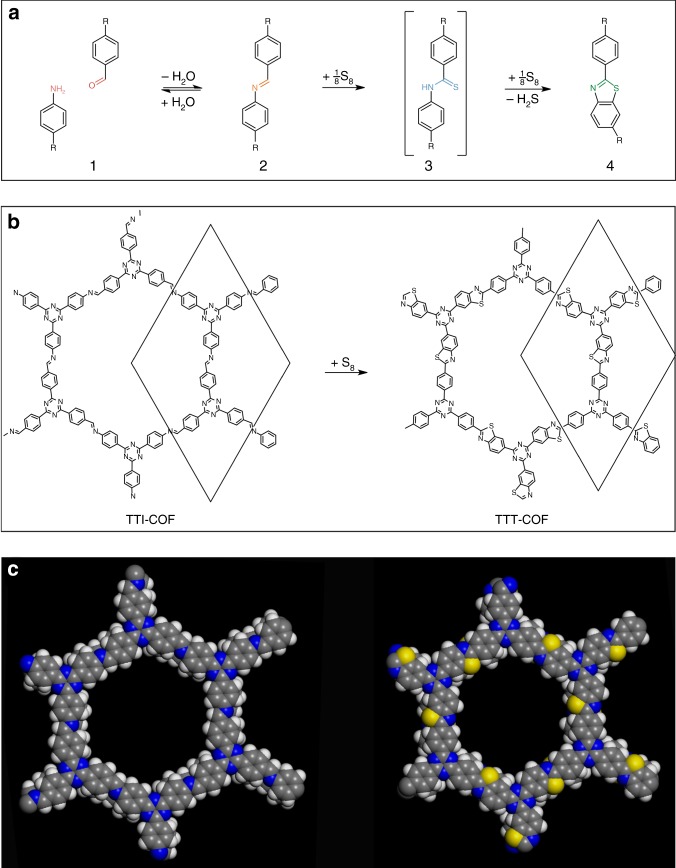
Schematic of the imine to thiazole transformation in the TTI-COF. **a** Schematic of the reaction of an amine and an aldehyde (1) to form an imine (2), then a thioamide as an intermediate (3) by the action of elemental sulfur, and finally a thiazole (4). **b** Schematic drawing of the sulfurization reaction of the TTI-COF to form the thiazole-based TTT-COF. **c** Space filling model of one pore of the TTI-COF (left) and the TTT-COF (right)

As pointed out by Yaghi et al.,^2^ the promise of COFs lies in the fact that COFs, though being extended solids, are amenable to the versatile toolbox of molecular synthesis. While this concept is particularly useful at the precursor level and hence formation of COFs, strategies for modifying the backbone of COFs once they are formed are extremely scarce^2^. We thus explored the post-synthetic reaction of COFs with elemental sulfur. At high temperatures, elemental sulfur reacts with aromatic imines to first oxidize the imine to a thioamide, and subsequently oxidatively cyclizes the thioamide group to form a thiazole ring (Fig. 1)^27^. Thus, sulfur serves as an oxidant (being reduced to H_2_S) and as a nucleophile, attaching first to the imine carbon and afterwards to the phenyl ring on the nitrogen side of the imine.

**Fig. 2:**
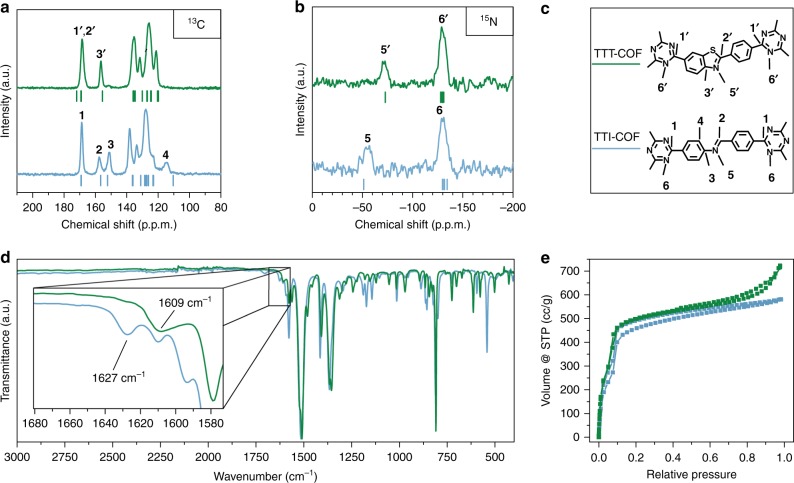
Characterization of the TTI-COF (blue) and TTT-COF (green). **a** ^13^C ssNMR demonstrating the conversion of the imine linkage to the corresponding thiazole. **b** ^15^N ssNMR showing a shift in the imine nitrogen position (**5** → **5′**). Calculated Δδ values for the TTT and TTI-COF on B97-2/pcS-2 level of theory are shown as red and black dashes, respectively. **c** Assignment of the ^13^C and the ^15^N ssNMR signals to the respective ^13^C and ^15^N nuclei in the structures. **d** FT-IR spectra of TTI-COF (black) and TTT-COF (red). The inset shows an enlargement of the region characteristic for N = C vibrations. **e** Argon sorption isotherms of TTI-COF and TTT-COF showing retention of porosity

Transferring this reaction scheme to imine-linked TTI-COF, which was previously reported by us to show high thermal and chemical stability^4,23^, we synthesized TTT-COF in two successive steps: First, TTI-COF was infiltrated with molten sulfur at 155 °C. At this temperature, sulfur has minimum viscosity, enabling mixing with the COF material. Subsequently, by using a thermal treatment at higher temperature (350 °C), the conversion of the TTI-COF to the TTT-COF took place. After removal of the excess sulfur by Soxhlet extraction and under high vacuum, the obtained material was investigated by ^13^C and ^15^N solid state NMR (ssNMR) to probe the imine to thiazole conversion and retention of the framework structure (Fig. 2).

### Framework characterization

As depicted in Fig. 2a, b, the ssNMR spectra of TTT-COF shows significant and well-defined changes as compared to the TTI-COF precursor. The loss of the carbon **3** and **4** signals at 151 ppm and 115 ppm (in TTI-COF) in the ^13^C ssNMR spectra, together with the appearance of the **3′** signal at 156 ppm for the TTT-COF, indicate the conversion of the nitrogen bearing phenyl ring to the thiazole in TTT-COF. Small residual intensity at 151 ppm might indicate some unreacted TTI-COF. Furthermore, the characteristic imine carbon **2** is shifted in the thiazole **2′** as a shoulder to the triazine carbon **1′**^28,29^. Also, the absence of a ^13^C signal between 210 and 180 ppm^29,30^ hints at the conversion of the imine to the thiazole and the absence of thioamide. ^15^N ssNMR shows the triazine nitrogen, **6** and **6′**, at the same position for TTT-COF as for TTI-COF and a shift of the imine nitrogen **5** from −55 ppm to the thiazole **5′** at −71 ppm. As additional confirmation of the determined thiazole structure of the TTT-COF, the calculated NMR chemical shifts of excised fragments (Fig. 2a, b, Supplementary Table 1, 2, 3, 4) are in good agreement with the experimental NMR spectra. ^13^C and ^15^N NMR chemical shifts were calculated with density functional theory (DFT) on the B97-2/3/pcS-2//PBE-D3/def2-TZVP level of theory (Supplementary Figs. 1, 2, 3, 4, 5), as we already applied this method successfully to other COF building blocks^31^.

The Fourier-transform infrared spectroscopy (FT-IR) spectra further confirm the conversion of TTI-COF to TTT-COF, as evident by the disappearance of the characteristic imine (N = CH) vibration at 1627 cm^−1^ and the appearance of a new N = C vibration^28^ of the thiazole at 1609 cm^−1^ (Fig. 2d, Supplementary Fig. 8). Elemental analysis shows the presence of sulfur with an elemental composition close to the composition that would be expected from the thiazole model (Supplementary Table 5). In addition, energy dispersive X-ray spectroscopy scanning electron microscopy (EDX/SEM; Supplementary Fig. 10) of TTT-COF indicates homogeneous distribution of sulfur in all parts of the sample, thus verifying a uniform and regular incorporation of sulfur in the COF backbone. The XRPD confirms the complete absence of reflections originating from elemental sulfur, further validating that only chemically bound sulfur is present (Supplementary Fig. 16).

Sorption analysis reveals retention of the porosity of the TTT-COF after sulfurization (Fig. 2e), the Brunauer–Emmett–Teller (BET) surface area of 1431 m^2^ g^−1^ for the TTT-COF (theoretical surface area 1609 m^2^ g^−1^) being close to the BET surface area of 1362 m^2^ g^−1^ for the precursor TTI-COF (theoretical surface area 1970 m^2^ g^−1^) (Supplementary Fig. 11). The ratio of experimental BET to theoretical surface area is seen to be improved upon sulfur incorporation. This not only indicates that the pores have not been blocked by sulfur deposits, but also that previously blocked pores might have been cleaned by oxidative or evaporative removal of guests in the pores. The pore size distribution was calculated from Argon isotherms using the quenched solid state functional theory (QSDFT) cylindrical pore model, which shows a reduction in the pore size from 2.3 nm in the TTI-COF to 2.2 nm in the TTT-COF (Supplementary Fig. 12). This change in the pore size matches well with the reduction in lattice parameters observed in the XRPD and the expected pore size reduction by the bending of the linkers upon formation of the thiazole.

### Structural analysis of TTI-COF and TTT-COF

**Fig. 3:**
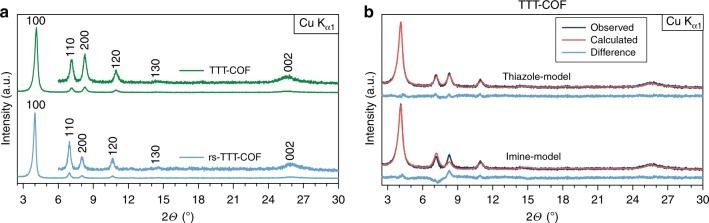
XRPD and modeling of the TTI- and TTT-COF. **a** XRPD patterns of the rs-TTI-COF and the TTT-COF showing retention of crystallinity upon transformation. **b** Comparison of the imine and thiazole models that were applied during Rietveld refinement of the XRPD TTT-COF. The thiazole model (top) shows a better fit than the imine model (bottom)

The structure and crystallinity of TTT-COF was then assessed with XRPD, revealing a crystalline material with a hexagonal unit cell (*P*6_3_/*m*) and a higher symmetry than derived for the precursor TTI-COF (*P*1) (Supplementary Fig. 13)^4^. However, TTT-COF is quite similar to the randomly stacked TTI-COF (rs-TTI-COF, *P*6_3_/*m*) (Fig. 3a), which is identical to the TTI-COF in terms of molecular connectivity, but shows random orientation of the stacking vector of the layers due to the altered synthesis conditions (see Methods section), and hence a higher apparent symmetry^4^. TTT-COF has reduced in-plane unit cell dimensions (24.478(5) Å vs 25.244(8) Å), and a larger interlayer stacking distance (*c* *=* 7.002(5) Å) than rs-TTI-COF (*c* *=* 6.905(7) Å) as is evident from Rietveld refinement (Supplementary Table 6, Supplementary Fig. 14). The smaller *a* and *b* axis of the unit cell can be understood by the contraction induced by bending of the linker upon formation of the five-membered thiazole ring (Supplementary Fig. 15). The larger stacking distance likely stems from the introduction of sulfur into the layers, also signaling a somewhat weaker interlayer interaction. This may explain the loss of the ordered slip-stacking that is present in TTI-COF (Supplementary Fig. 13). Rietveld refinement of the XRPD pattern of TTT-COF was done using an imine model and a thiazole model; a significantly better fit was obtained for the thiazole model, which further confirms this structural feature in TTT-COF (Fig. 3b, Supplementary Table 7).

### Generalizing the imine locking with a pyrene based COF

To demonstrate that the concept of imine to thiazole conversion in COFs can be generalized and transferred also to other COF systems, we performed the reaction on the Pyrene tetra(phenyl) biphenyl imine-COF (PBI-COF, Supplementary Fig. 6)^32^, which was transformed to the Pyrene tetra(phenyl) biphenyl thiazole-COF (PBT-COF, Supplementary Fig. 6). In the PBI- and PBT-COFs the conversion is clearly evidenced by ^13^C ssNMR as well, which shows the appearance of two peaks at 153.3 ppm and 166.8 ppm, with the latter corresponding to the characteristic thiazole carbon between the nitrogen and the sulfur (Supplementary Fig. 7). Note that in the TTI- and TTT-COFs, this region is obstructed by the presence of the triazine carbon (**1** & **1′**, Fig. 2a). Similar to the TTI- to TTT-COF conversion, the PBI to PBT-COF conversion is evidenced by the disappearance of the vibration at 1622 cm^−1^ that corresponds to the characteristic imine stretch, and by the presence of a vibration at 1602 cm^−1^ in the PBT-COF that can be assigned to the thiazole moiety (Supplementary Fig. 9).

As in the TTI-to-TTT transformation, crystallinity was retained during the transformation of the PBI- to PBT-COF (Supplementary Fig. 17), while the in-plane lattice parameters *a* and *b* of the Rietveld-refined structures differ less between PBI- and PBT- COF (Supplementary Table 8) as compared to the TTI- and TTT-COF (0.60 Å and 1.58 Å for the PB and the TT system, respectively). This effect is attributed to the lower degree of structural distortion during the sulfurization reaction, as seen in Supplementary Fig. 18.

### Chemical stability screening

**Fig. 4:**
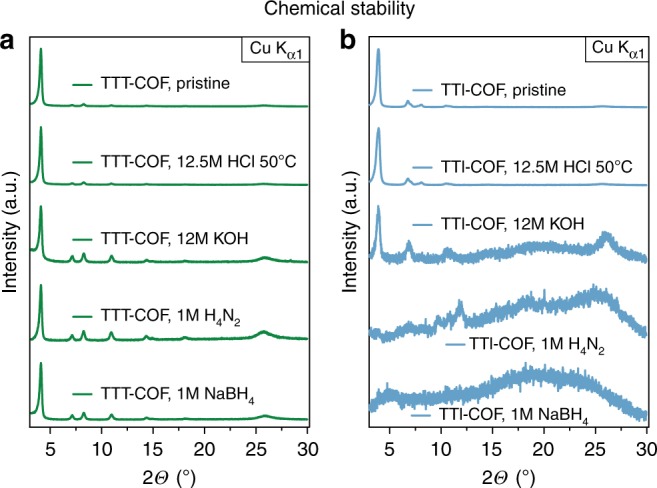
Chemical stability tests of the TTI- and TTT-COFs. Stability is judged by the crystallinity of the frameworks after exposure to various aggressive chemicals. The TTI- and TTT-COF samples were each exposed to identical conditions for 16 h, which showed a substantially higher stability of the TTT-COF (**a**) against reactive conditions than the corresponding TTI-COF (**b**)

As the transformation of the imine-based TTI-COF to the thiazole-based TTT-COF is expected to significantly improve the chemical stability, we assessed the possibility of locking the reversible bond by comparing the crystallinity before and after chemical treatment. Both materials were exposed to identical and extremely harsh conditions, to test the limits of stability of both COFs (Fig. 4). Initially both COFs were treated with concentrated hydrochloric acid, after which both COFs showed no signs of degradation, testifying to the already excellent resistance to acids of TTI-COF. In contrast, other imine-based COFs have previously been reported to be labile under strongly acidic conditions^2^. The treatment of TTI-COF with potassium hydroxide solution lead to a near complete loss of crystallinity, while the TTT-COF remained unaffected. Next, we tested reagents that are known to alter imine bonds: hydrazine is a particularly good nucleophile that enters the imine bond and replaces the amine, which then leads to a loss of order; sodium borohydride, a reagent that is used to reduce imine bonds could lead to a loss of rigidity, followed by a collapse of the structural order. In both cases the TTT-COF remains essentially unaffected, while the TTI-COF turns completely amorphous. This result shows that the imine bond has been locked as a thiazole, while the ordered structure of the TTT-COF is retained. The resilience of the TTT-COF to reactive conditions and reagents could enable a range of applications that were previously not accessible due to the lability of COFs.

### Real structure and defect analysis by TEM

TEM investigations in COFs have so far primarily been used to confirm the periodic structure of COFs^4,11,23,33,34^, their nano- and micron-scale morphology^12^, and the presence of inorganic guests^33,35^. However, the low contrast in COFs and their low electron beam stability generally render a detailed analysis of COFs with TEM highly challenging as the samples easily decompose before useful information can be extracted. The destruction of COFs in the electron beam can be ascribed to different damaging mechanisms: one of the defining features of COFs is their composition out of light elements, which makes them difficult to image since such atoms can be significantly affected by atom displacement, electron-beam sputtering, electron-beam heating, electrostatic charging, and radiolysis^36^.

**Fig. 5:**
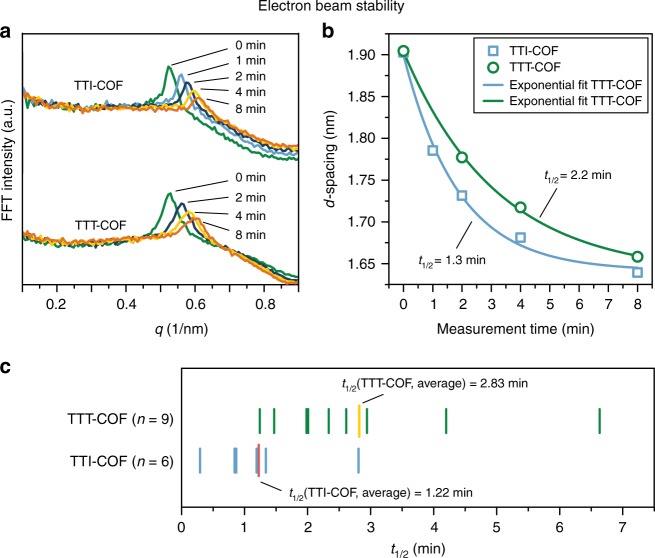
Electron beam stability tests of the TTI- and TTT-COFs. **a** Electron beam damage seen by the broadening and a shift of the peak in the FFT, corresponding to the 100 reflection in XRPD, of TEM images taken after different exposure times. **b** Plotting the exposure time against the peak shift in the FFT shows a decay that can be fitted with an exponential (fit parameters shown in Supplementary Table 10). **c** Several sets of images were analyzed this way and the results are depicted graphically for better comparison. The average half-life of each system reveals the higher stability of the TTT-COF as compared to TTI-COF

The TTI- and TTT-COF were analyzed by electron microscopy, where TEM and SEM revealed a morphology of inter-grown crystallites with a slightly anisotropic shape in both COFs (Supplementary Fig. 19). Imaging TTI- and TTT-COF by TEM suggests significantly increased electron contrast for TTT-COF and improved electron beam stability as compared to TTI-COF and other COFs^4,33^. First, we quantified the stability of the TTI- and the TTT-COF in the electron beam by taking images at defined time intervals, under otherwise identical conditions. Visual inspection of the images revealed gradual decomposition of the TTI-COF upon electron beam exposure as evident by shrinking of the structure as well as diminished lattice fringes (Supplementary Fig. 20). Quantitative analysis of these images by means of their fast Fourier transform (FFT) showed a continued broadening and shift to smaller *d*-spacings for the peak corresponding to the 100 reflection in the XRPD. The continuous shift in *d*-spacing can be fitted by an exponential decay from which half-lives can be extracted. TTI-COF has an average half-life of 1.22 min, while TTT-COF displays a significantly increased half-life in the electron beam of 2.83 min (Supplementary Fig. 21), thus clearly pointing to the higher stability of TTT-COF in the electron beam (Fig. 5). While the higher electron contrast in the TEM images of the TTT-COF results from the regular incorporation of sulfur into the lattice, the improved stabilization is likely due to the aromatization of the imine bond in the form of a thiazole and the lower number of hydrogen atoms in the structure, which are most susceptible to electron beam damage^36^. This improved stability is crucial for exploring the real structure of the TTT-COF with TEM as described in the following.

**Fig. 6:**
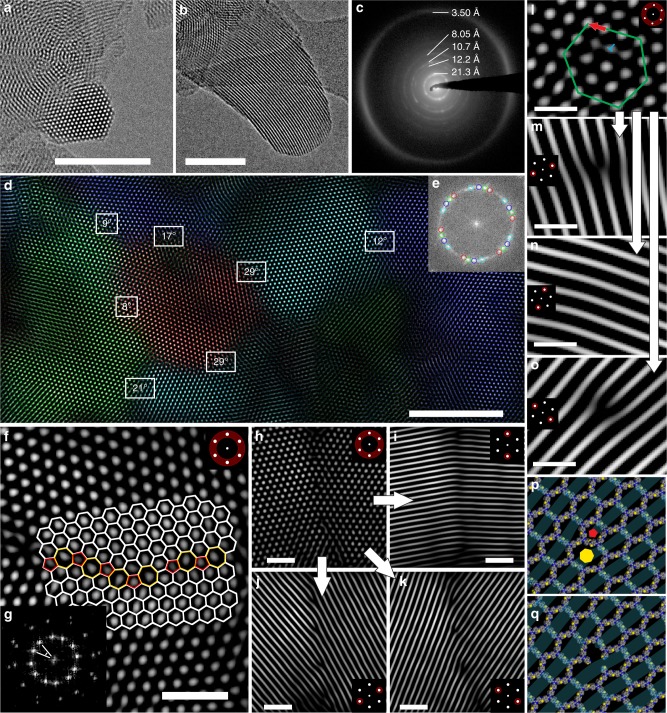
TEM images and TEM analysis of TTT-COF. FFT filters applied to the image are indicated by a schematic inset. **a**, **b** Individual crystallites in different orientations. **c** SAED with logarithmic contrast showing diffraction rings, which are in agreement with the XRPD pattern. **d** Multiple inter-grown grains visible by TEM. Indicated angles show the relative orientation of neighboring crystallites. Color overlay indicating the individual grains generated by applying selective hexagonal Fourier filtration (**e**). **f** High angle grain boundary of crystallites with co-aligned *c* direction with an overlay indicating the interface consisting of five, six and seven membered rings. **g** FFT of image **f**. **h**–**k** Low angle grain boundary with different Fourier filters applied, visualizing the starting points of edge dislocations. **l** Close-up of the start of an edge dislocation. The beginning of the edge dislocation is indicated by the blue **T**, the Burgers vector (red arrow) is determined from the green hexagon to be [100]. **m**–**o** Visualization of the edge dislocation position from image **l** with different Fourier filters. **p**, **q** Modeling of an edge dislocation in Materials Studio utilizing a screw dislocation along the pentagonal (red pentagon) and the heptagonal channel (yellow heptagon) (**p**) and the edge dislocation visualized as a channel linker vacancy (**q**). Scale bars: **a**, **b**, **d**: 50 nm; **f**, **h**–**k**: 10 nm; **l**–**o**: 5 nm

TEM images of TTT-COF reveal an overall retention of the crystallite size compared to the precursor TTI-COF (50–200 nm); likewise, the crystallinity seen already for the TTI-COF is clearly retained as well^4^. The hexagonal symmetry of the structure is visible from the real space images (along the [001] zone axis, Fig. 6a), which also show the presence of continuous pore channels when viewed along [*hk*0] (Fig. 6b). Both real space images and selected area electron diffraction (SAED) patterns (Fig. 6c) are in agreement with the structural model developed with Rietveld refinement (see also Supplementary Figs. 22, 23, 24).

In addition, TEM reveals a host of real structure details of TTT-COF, including many forms of defects such as disorder, twin, and grain boundaries. Several observed grains of the COF have co-aligned *c*-axes but are rotated against each other in the *ab*-plane (Fig. 6d). Two main types of boundaries are visible between grains: high angle grain boundaries (HAGB) and low angle grain boundaries (LAGB). HAGB are formed by a corrugated interface of adjacent five and seven membered rings in contrast to the normal six membered rings in the ordered TTT-COF, as shown in Fig. 6f. Some crystallites have an angle of 29° between them, previously observed by TEM in covalently connected grain boundaries of single-layer graphene showing quilt-like structures^37^, similar to the co-aligned crystallites of TTT-COF. It was not possible to discern unambiguously whether a covalent interface exists between the grains due to increased radiation damage at higher magnifications. However, having this many different crystallites in such an oriented way and intimate contact between the different domains suggests an alignment of these domains during synthesis, which is likely induced by covalent connections.

## Discussion

**Fig. 7:**
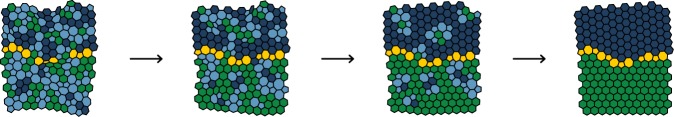
Schematic of the proposed grain boundary formation mechanism. An initially amorphous gel slowly crystallizes to form the interface between two grains, which implies covalent connectivity between grains as a remnant of the amorphous state

A likely mechanism for the formation of the observed grain boundaries is the crystallization of grains from an initially amorphous imine gel (Fig. 7) as has been proposed as a formation mechanism for imine COFs by Dichtel and coworkers^38^. This mechanism implies that the covalent connectivity between the different grains is present from the initial formation of the polymer on and that the grain boundary is left as a remnant of this amorphous state.

The intergrowth is even more likely for LAGB as these present a nearly continuous transition from one crystallite to another. Inspection of the LAGB with different Fourier filters shows the presence of several edge dislocations (Fig. 6h–k). Details of such an edge dislocation with a Burgers vector of [100] are shown in Fig. 6l–o. At the molecular level, such a defect could be described by either a linker vacancy (Fig. 6p–q, and supplementary discussion) or an out-of-plane growth that resembles five- and seven-membered rings such as spirals. Features such as five membered rings and the growth of spirals have similarly been described in the simulation of the crystallization of COF-5, thus predicting the presence of these features in a COF, which have been observed here^39^.

The observed defects in TTT-COF might have important implications for the properties of the COF. The grain boundaries of the co-aligned crystallites would not obstruct the pore channels and therefore are not expected to influence properties that are primarily contingent on the porous nature of COFs, such as their use as a sorption material or membranes where continuous mass transport is important. The electronic or excitonic conductivity in COFs is assumed to require ordered π-stacking for charge carrier percolation perpendicular to the layers^15^, while the transport of charges is also possible within the *ab*-plane of the individual layers^40^. In the latter case the covalent connection and co-alignment of the COF layers could still enable charge transport from one grain to another, rendering limitations through reduced grain boundary conductivity less severe. The presence of defects extending beyond one layer such as out-of-plane helices would essentially turn the 2D COF into a covalently connected 3D COF (Supplementary Fig. 26). This could have important implications for the feasibility of exfoliation of nominal 2D COFs, as covalent bonds would need to be broken in order to separate the individual “layers” of the COF. The presence of an isolated vacancy or a columnar vacancy line defect in the COF structure would influence not only the sorption properties of the COF by the presence of differently sized pores, but it would also present functional groups exposed to larger than regular pores in the COF. Furthermore, we note that size-selective properties such as sorption and catalysis^41^ would be influenced in terms of selectivity by the presence of defects, again emphasizing the importance of real structure effects for the properties of COFs.

The reaction of the TTI-COF with sulfur proceeds under conditions that should not allow opening of the imine bonds; the reaction is performed in neat sulfur and no water is present. Furthermore, special solvent mixtures are required to form a porous crystalline COF while reversible bonds are formed and broken. Since these conditions are not met during the sulfurization reaction described, the formation of TTT-COF has to happen in a topochemical fashion with minimal structural disruption of the covalent molecular backbone and the retention of the hexagonal structure of the material. While oxidative conditions during the thiazole formation might cause the scission of the covalent backbone, it cannot explain the presence of the observed defects such as grain boundaries and edge dislocations. We thus note that the observed defects in TTT-COF have to be present already in TTI-COF and likely in other COFs as well, especially 2D COFs based on the same topology.

In summary, the reaction of an imine COF with elemental sulfur leads to the topochemical formation of aromatic thiazole moieties as a robust linkage group, which causes a change in the symmetry of the COF crystal, but not of its topology or its connectivity. This reaction therefore adds to the synthetic toolbox of post-synthetic locking of COFs, which helps to circumvent the inherent limitations imposed by the presence of reversible bonds. The effect of this locking strategy was exemplified by the significantly improved chemical stability of the resulting thiazole COF. In addition, sulfur-assisted generation of thiazoles increases the number of possible COF structures and at the same time opens the door to new COFs with chemical properties not attainable in materials synthesized by reversible reactions.

While crystallinity and porosity of the TTT-COF are fully retained, it shows significantly improved electron contrast compared to the parent COF in addition to improved stability to the electron beam, thus making this system amenable to a study of its real structure by TEM. Close inspection of the structure of the TTT-COF allowed us to extract valuable information on both its long-range and local structure, including the nature of defects and disorder present in the system. While locked in during TTT-COF formation, these defects have been introduced already during the (reversible) synthesis of the precursor TTI-COF and thus can be considered as lasting fingerprints of the COF formation process. In particular, we find prevalent one-dimensional defects such as edge dislocations as well as co-aligned COF grains with grain boundaries that are likely covalently connected. Unraveling the nature of defects in COFs is not only key to better understand their impact on the optical, electronic and catalytic properties of COFs, but also to control and design new COFs by targeting properties imbued by such defects.

## Methods

### Synthesis

Triazine triphenyl thiazole COF (TTT-COF) and Pyrene tetra(phenyl) biphenyl thiazole (PBT-COF): The respective imine COF was activated under high vacuum at 150 °C and subsequently mixed with the 15-fold amount (by weight) of sulfur in a ball mill. The resulting homogeneous mixture was transferred to a quartz boat in a horizontal tubular furnace and purged at 60 °C under flowing argon. The temperature was increased to 155 °C (60 K h^−1^ heating rate) and maintained there for 3 h. Subsequently, the temperature was raised to 350 °C (100 K h^−1^ heating rate) and kept for 3 h. After cooling down, the resulting material was washed via Soxhlet extraction with toluene and THF for 24 h, respectively. The samples were dried at 70 °C in an oven and then at 150 °C under high vacuum.

Randomly stacked Triazine triazine triphenyl imine COF (rs-TTI-COF): TT-CHO (0.0254 mmol, 10.0 mg), TT-NH2 (0.0254 mmol, 9.01 mg), di(n-octly)phthalate (1 ml), triphenyl phosphate (1 ml), aqueous acetic acid (0.318 mmol, 6 M, 0.053 ml) were added successively to a Biotage^©^ precision glass vial, sealed and heated under autogenous pressure at 120 °C for 72 h. The rs-TTI-COF was worked up in the same manner as TTI-COF.

### Chemical stability tests

Chemical stability of the TTI- and TTT-COF was assessed by immersing ~20 mg of the COF in an aqueous solution of each 12.5 M hydrochloric acid (HCl) (50 °C), 12 M potassium hydroxide (KOH), 1 M hydrazine (H_2_NNH_2_) and 1 M sodium borohydride (NaBH_4_) for 16 h at room temperature unless denoted otherwise. Afterwards, the sample was filtered off and washed thoroughly with water, ethanol, THF, chloroform and DCM. After drying at ambient conditions, the crystallinity was assessed by XRPD.

### Structure building

The structural models were built successively and based on each other starting from the well defined TTI-COF model^4^. The rs-TTI-COF and the TTT-COF showed no symmetry reduction from a hexagonal to a triclinic unit cell, therefore the highest reasonable symmetry supported by the molecular geometry is *P*6_3_/*m* which was used to build a unit cell model in BIOVIA Materials Studio 2017 (17.1.0.48. Copyright © 2016 Dassault Systèmes). Molecular connectivity was based on geometric considerations and the obtained evidence from FT-IR and ssNMR. The structures and the unit cell was relaxed using force fields (Forcite, universal force fields with Ewald electrostatic and van der Waals summations method). These models were then used to refine the unit cell parameters by Rietveld refinement.

### TEM and SAED

TEM was performed with a Philips CM30 ST (300 kV, LaB_6_ cathode). The samples were suspended in *n*-butanol and drop-cast onto a lacey carbon film (Plano). Processing of TEM and SAED images was performed with the help of ImageJ 1.47 v.

Stability measurements were performed by taking images of the sample after defined time intervals in-between pictures, relative to the first image (*t* = 0 min).

### XRPD

XRPD patterns were collected on a Stoe Stadi P diffractometer (Cu–K_α1_, Ge(111)) in Debye-Scherrer geometry. The sample was measured inside a sealed glass capillary (1.0 mm) that was spun for improved particle statistics.

The powder patterns were analyzed by Rietveld^42^ and Pawley^43^ refinement using the range from 2–30° 2*θ* with TOPAS V5, while keeping the atom coordinates fixed. The peak profile was described by applying the fundamental parameter^44^ approach as implemented in TOPAS. The background was modeled with a 6th order Chebychev polynomial. Lattice parameters were refined as constrained by the symmetry. The peak broadening was modeled with asymmetry adopted phenomenological model for microstrain^45^. The plotted XRPD patterns were normalized to compare relative peak intensities.

### ssNMR

The ssNMR spectra were recorded on a Bruker Avance III 400 MHz spectrometer (B_0_ = 9.4 T) at the frequencies of 400.1, 100.6 and 40.8 MHz, for ^1^H, ^13^C and ^15^N, respectively. The ssNMR experiments were performed on a Bruker double resonance 4 mm MAS probe with the COF samples packed in ZrO_2_ rotors. The ^1^H-^13^C and ^1^H-^15^N cross-polarization (CP) MAS spectra were recorded with a rotation frequency of 10–12 kHz using a ramped-amplitude (RAMP) spin-locking pulse on the proton channel. The contact time for both nuclei was set to 5 ms, which was found to be optimal. The recycle delay in the CP-experiments was 2 s, defined primarily by the spin-lattice relaxation of protons. All solid-state experiments were carried out using SPINAL64 composite-pulse proton decoupling with radio frequency power between 70 and 80 kHz. The reported ^1^H and ^13^C chemical shifts were referenced to tetramethylsilane (TMS), while the ^15^N shifts were referenced to nitromethane.

### Quantum‐chemical calculations

Atom positions and lattices of periodic structures were optimized on PBE-D3/def2‑TZVP^46,47^ level of theory using an acceleration scheme based on the resolution of the identity (RI) technique and the continuous fast multipole method (CFMM)^48^ in a developer version of Turbomole^49^.

The CFMM uses multipole moments of maximum order 20 together with a well-separateness value 3 and a basis function extent threshold of 10^-9^ a.u. Grid 7 was used for the numerical integration of the exchange-correlation term. The norm of the gradient was converged to 10^-4^ a.u. and the total energy is converged to 10^-8^ Hartree within the structure optimization using an equidistant 5 × 5 k-point grid.

NMR chemical shifts were obtained on B97-2/pcS-2//PBE-D3/def2-TZVP level of theory^46,47,50,51^ using the Turbomole program package in version 7.0.2 for geometries and the FermiONs++^52,53^ program package for the calculation of NMR chemical shifts performed on excised sections (Supplementary Figs. 3, 4; distinction shown in Supplementary Fig. 5) of the TTT- and TTI-COF models. Chemical shifts were then referenced to the experimentally obtained spectra with the triazine peak 1/1′ and 6,6′.

### Data availability

All relevant data are available from the authors upon reasonable request.

## Electronic supplementary material

Supplementary Information
