# Supplementary material for: Topochemical conversion of an imine- into a thiazole-linked covalent organic framework enabling real structure analysis
Source: Nat Commun. 2018 Jul 3;9:2600. doi: 10.1038/s41467-018-04979-y (PMC6030076; doi:10.1038/s41467-018-04979-y)
Supplement: Supplementary file 1 — Supplementary Information [file 41467_2018_4979_MOESM1_ESM.pdf]

Supplementary Information for:

Topochemical conversion of an imine-into a thiazole-linked covalent organic framework enabling real-structure analysis

Haase et al.

Supplementary Methods

Materials

*T*ris(4-formylphenyl)*t*riazine (TT-CHO),[1] tris(4-aminophenyl)triazine (TT-NH2),[1] tetra(4-formylphenyl)pyrene[2] were synthesized according to literature procedures. All other chemicals were obtained from commercial sources.

Synthesis

TTI-COF was synthesized as previously described.[1,3] TT-NH2 (0.0635 mmol, 22.5 mg), TT-CHO (0.0635 mmol, 25.0 mg), 1,4-dioxane (2.5 ml), mesitylene (2.5 ml), aqueous acetic acid (0.794 mmol, 6M, 0.132 ml) were added to a Biotage© precision glass vial, sealed and heated under autogenous pressure at 120°C for 72 h. After the reaction was allowed to cool down, the reaction mixture was filtered and washed thoroughly with ethanol, water, tetrahydrofuran and chloroform and then dried in high dynamic vacuum overnight.

*P*yrene tetra(phenyl) *b*iphenyl *i*mine PBI-COF was synthesized from tetra(4-formylphenyl)pyrene and diaminobiphenyl according to the literature procedure. [4]

SEM/EDX

SEM SE (secondary electron) detector images were obtained on either a Zeiss Merlin or a VEGA TS 5130MM (TESCAN) with a SEM-EDX using a Si/Li detector (10kV acceleration voltage, Oxford).

FT-IR

Infrared spectra were recorded in attenuated total reflection (ATR) geometry on a PerkinElmer UATR Two equipped with a diamond crystal.

Sorption

Sorption measurements were performed on a Quantachrome Instruments Autosorb iQ MP with Argon at 87K. The pore size distribution was determined from argon adsorption isotherms using the QSDFT cylindrical pores in carbon model for argon at 87 K.

Elemental analysis

CHNS elemental analyses were performed with a Vario EL elemental analyser (Elementar Analysensysteme GmbH).

Supplementary Table 1: Calculated TTT-COF ^{15}N-NMR chemical shifts.

		NMR Chemical Shift [ppm]			
**Atom Label**	**Atom**	**TTT-Section 1**	**TTT-Section 2**	**TTT-Section 3**	**Average**
1 (6‘)	N	-131.35	-130.90	-133.51	-131.92
10 (5‘)	N	-76.67	-76.66	-76.72	-76.68
18 (6‘)	N	-136.41	-134.18	-134.37	-134.99
33 (6‘)	N	-134.06	-134.64	-132.60	-133.77
38 (6‘)	N	-134.29	-131.80	-133.79	-133.29

Shifts were calculated for three distinct TTT-COF sections (Supplementary Figure 3, Supplementary Figure 4, Supplementary Figure 5) on B97-2/def2-TZVP level of theory.

Supplementary Table 2**:** Calculated TTT-COF ^{13}C-NMR NMR chemical shifts.

		NMR Chemical Shift [ppm]			
**Atom Label**	**Atom**	**TTT-Section 1**	**TTT-Section 2**	**TTT-Section 3**	**Average**
2 (1‘)	C	179.89	180.64	179.36	179.96
3	C	146.95	146.97	146.92	146.95
4	C	138.17	138.17	138.09	138.14
5	C	135.46	135.43	135.46	135.45
6	C	145.62	145.69	145.57	145.63
7	C	135.65	135.67	135.71	135.67
8	C	137.88	138.00	137.90	137.92
9 (2‘)	C	182.95	182.98	183.01	182.98
11 (3‘)	C	166.48	166.51	166.56	166.52
12	C	146.54	146.55	146.52	146.54
13	C	131.31	131.47	131.40	131.39
14	C	141.05	141.09	141.10	141.08
15	C	135.73	135.70	135.80	135.74
16	C	130.85	130.85	130.85	130.85
17 (1‘)	C	179.59	180.27	180.90	180.25

Shifts were calculated for three distinct TTT-COF sections (Supplementary Figure 3, Supplementary Figure 4, Supplementary Figure 5) on B97-2/def2-TZVP level of theory.

Supplementary Table 3: Calculated TTI-COF ^{15}N-NMR chemical shifts.

Atom Label	Atom	TTI-Section 1	TTI-Section 2	TTI-Section 3	Average
		NMR Chemical Shift [ppm]			
8 (5)	N	-50.84	-51.68	-51.03	-51.18
16 (6)	N	-130.23	-129.46	-129.36	-129.68
26 (6)	N	-128.71	-131.14	-130.75	-130.20
38 (6)	N	-134.33	-134.14	-134.88	-134.45
39 (6)	N	-131.81	-131.58	-131.87	-131.75

Shifts were calculated for three distinct TTI-COF sections on B97-2/def2-TZVP level of theory.

Supplementary Table 4: Calculated TTI-COF ^{13}C-NMR NMR chemical shifts.

Atom Label	Atom	TTI-Section 1	TTI-Section 2	TTI-Section 3	Average
		NMR Chemical Shift [ppm]			
9 (3)	C	164.28	164.13	164.40	164.27
10 (4)	C	122.39	122.32	122.43	122.38
11	C	139.64	139.71	139.71	139.69
12	C	143.12	143.20	142.95	143.09
13	C	139.42	139.38	139.34	139.38
14	C	134.95	135.14	134.86	134.98
15 (1)	C	181.01	181.11	180.82	180.98
27 (1)	C	181.75	180.50	181.14	181.13
28	C	148.13	148.00	148.23	148.12
29	C	138.41	138.28	138.33	138.34
30	C	135.31	135.25	135.29	135.29
31	C	148.44	148.35	148.29	148.36
32	C	140.50	140.69	140.55	140.58
33	C	138.17	138.22	138.27	138.22
34 (2)	C	168.63	168.57	168.68	168.62

Shifts were calculated for three distinct TTI-COF sections on on B97-2/def2-TZVP level of theory.

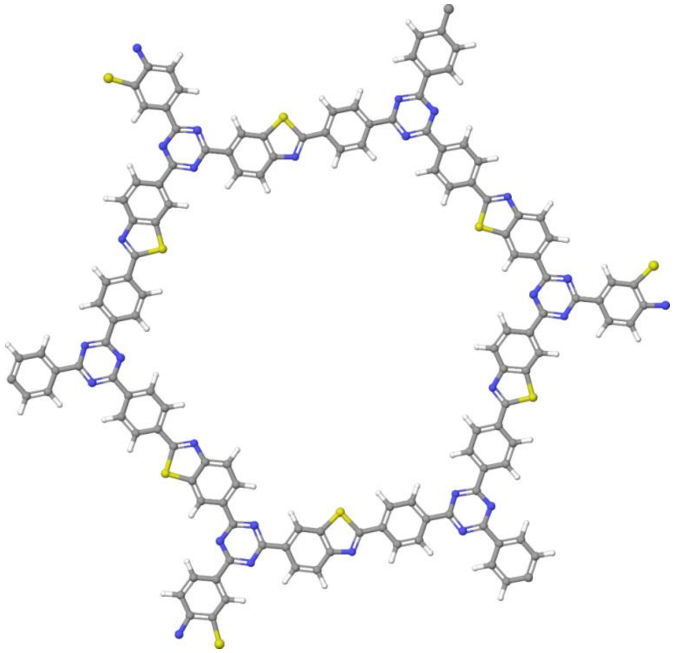

Supplementary Figure 1**:** Optimized geometry for a single TTT-COF pore. The structure was excised from a 2x2 supercell obtained from periodic calculations on PBE-D3/def2-TZVP level of theory using 5x5 k-points.

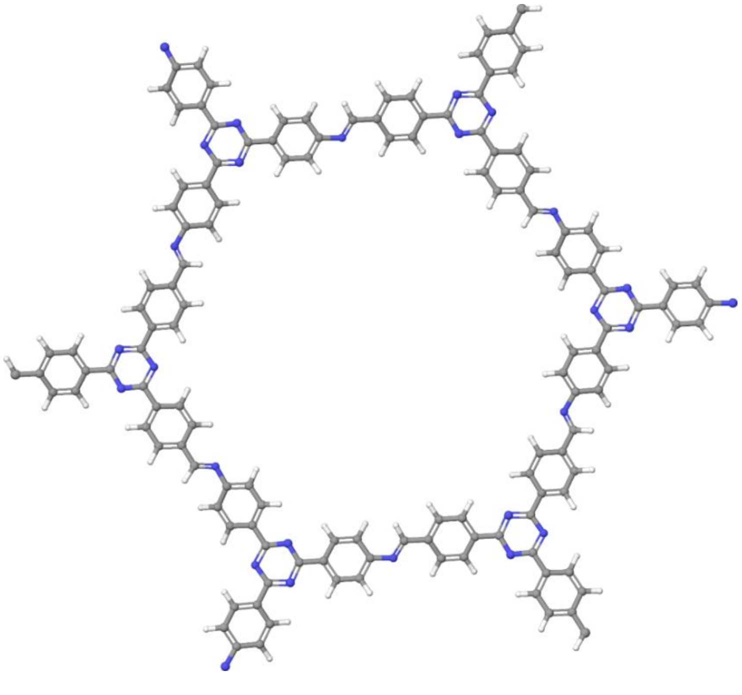

Supplementary Figure 2**:** Optimized geometry for a single TTI-COF pore. The structure was excised from a 2x2 supercell obtained from periodic calculations on PBE-D3/def2-TZVP level of theory using 5x5 k-points.

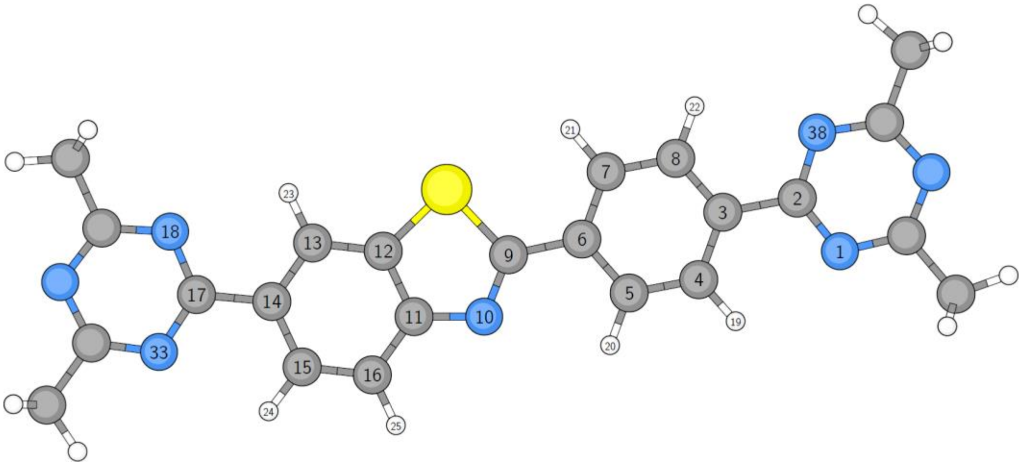

Supplementary Figure 3**:** Atom labels for the excised TTT-Sections. The structural model is based on the optimized geometry of the COF.

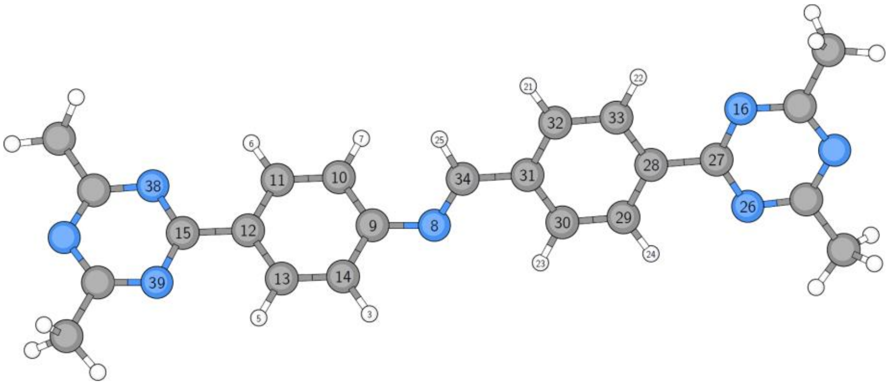

Supplementary Figure 4**:** Atom labels for the excised TTI-Sections. The structural model is based on the optimized geometry of the COF.

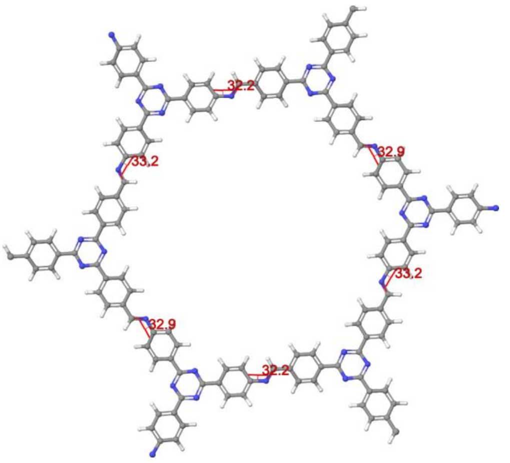

Supplementary Figure 5**:** Dihedral angles in a single TTI-COF pore. Dihedral angles for all imine bonds within the optimized geometry of a single TTI-COF ring, identifying three distinct sections: Section-1, 32.9°; Section-2, 32.2°; Section-3, 33.2°. Corresponding sections were also selected for the TTT-COF pore.

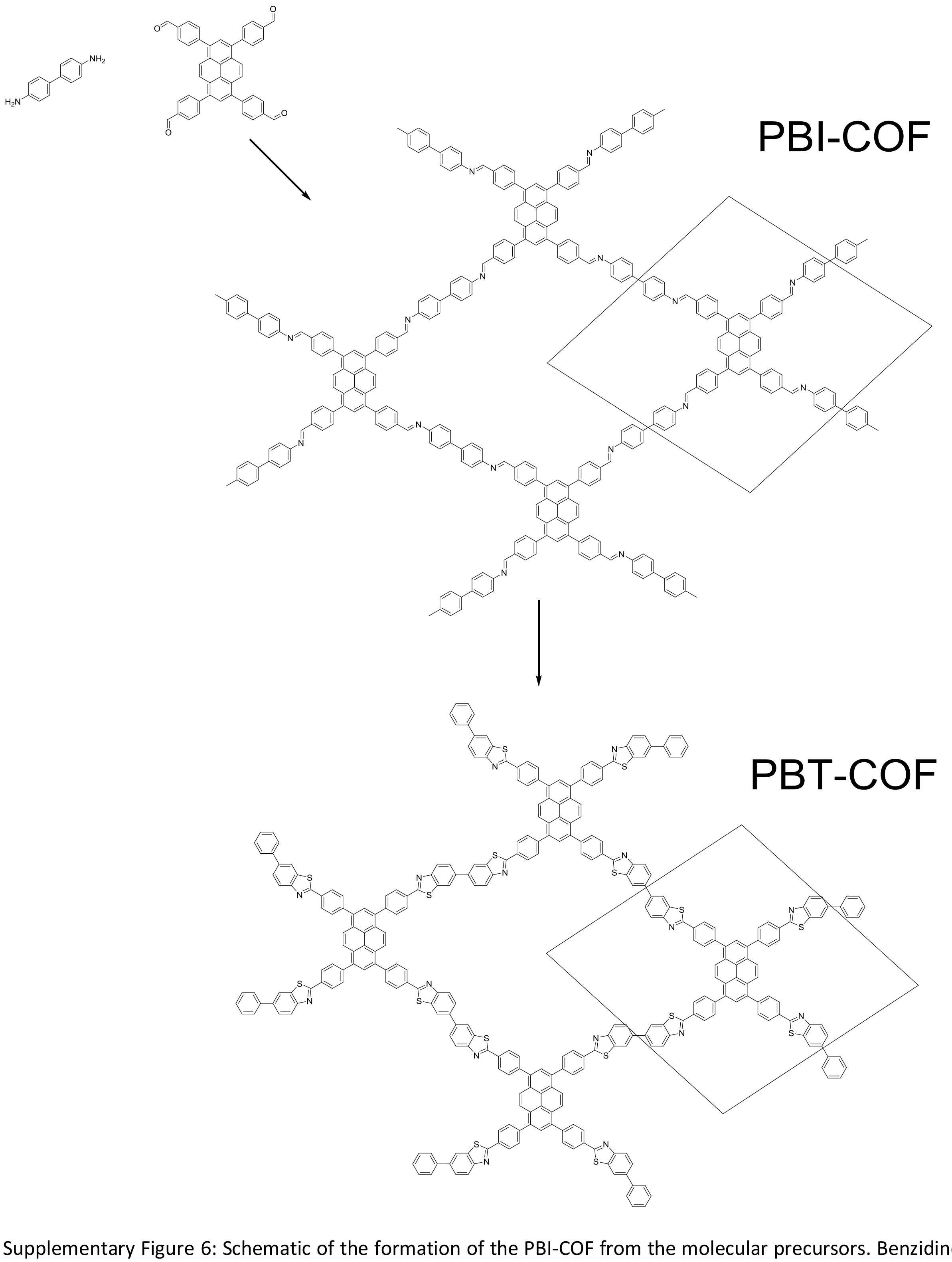

Supplementary Figure 6: Schematic of the formation of the PBI-COF from the molecular precursors. Benzidine and tetra(formylphenyl) pyrene react under reversible conditions to form the PBI-COF. The PBI-COF is then transformed into the PBT-COF by reaction with sulphur.

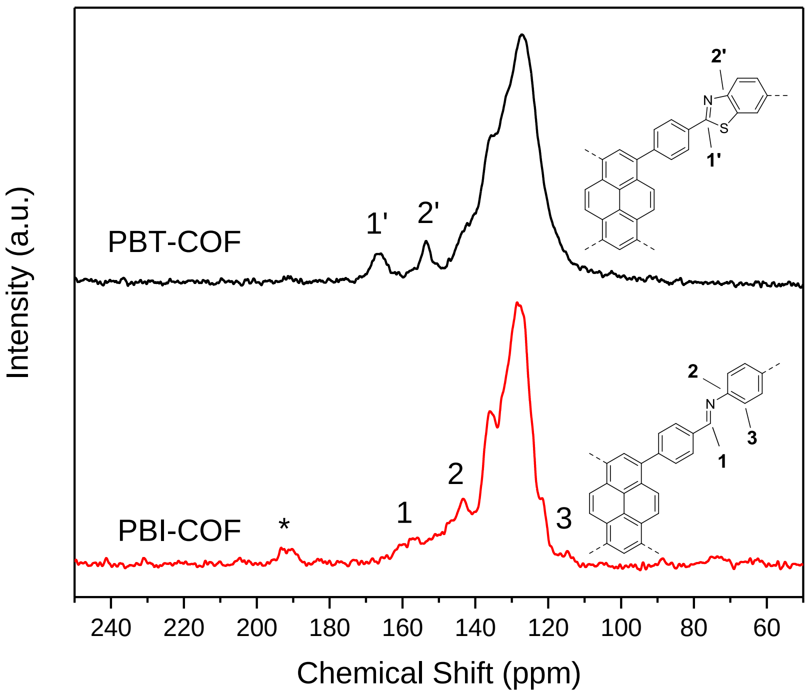

Supplementary Figure 7: ^{13}C ssNMR of the PBI-COF and PBT-COF. Tentative assignment of the characteristic peaks is shown. The peak indicated by the asterisk points towards residual aldehyde left from the COF synthesis, which is gone after the sulphur incorporation.

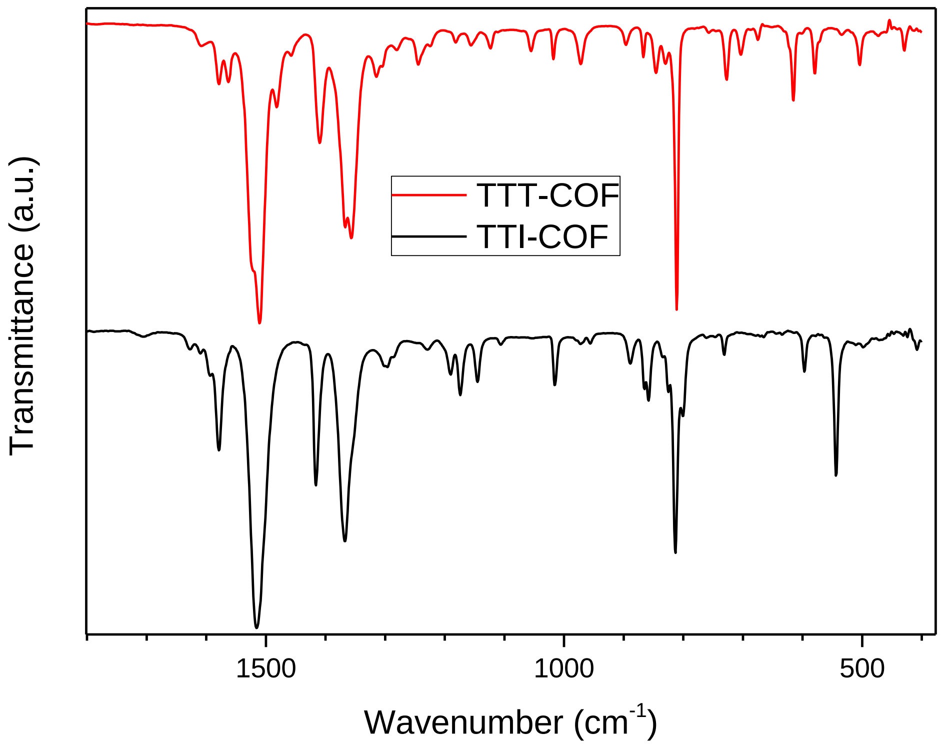

Supplementary Figure 8: Zoomed section of the IR spectra of the TTI- and TTT-COF. The IR shows significant changes between the imine and the thiazole-based COFs.

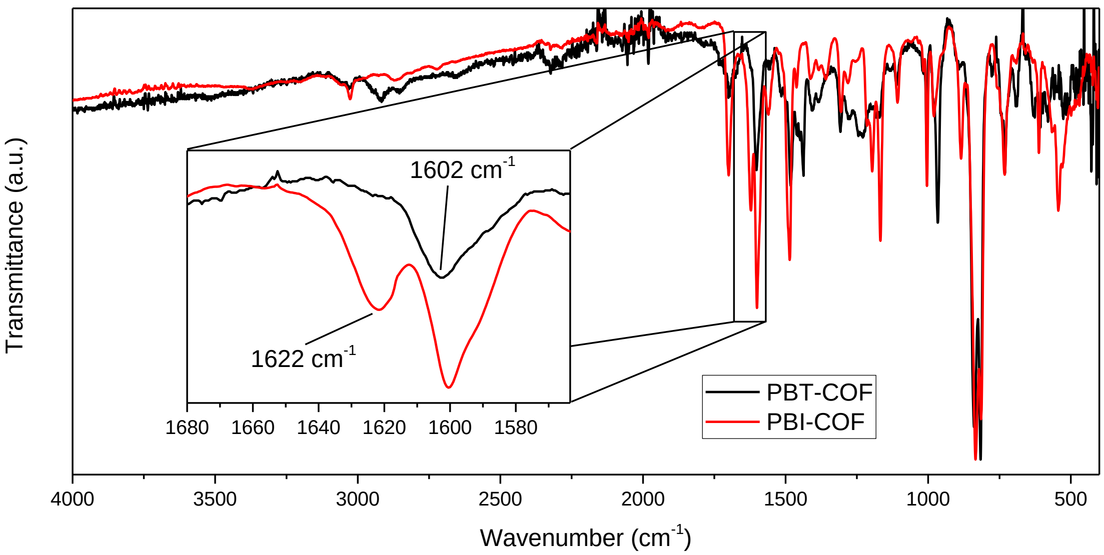

Supplementary Figure 9: IR spectra of the PBT- and the PBI-COF. The enlarged inset shows the region of the characteristic imine vibration (1622 cm^{-1}) in the PBI-COF that is lost upon transformation to the thiazole. The corresponding thiazole vibration can be assigned to the band at 1602 cm^{-1}.

Supplementary Table 5: Elemental analysis of the TTT-COF and the TTI-COF.

**TTT-COF**	**C**	**N**	**H**	**S**
**Measured**	66.09 (10)	15.11 (17)	2.68 (14)	13.56 (22)
**Expected**	68.95	16.08	2.70	12.27

**TTI-COF[1]**	**C**	**N**	**H**	**S**
**Measured**	76.85	18.37	3.79	-
**Expected**	77.91	18.17	3.92	-

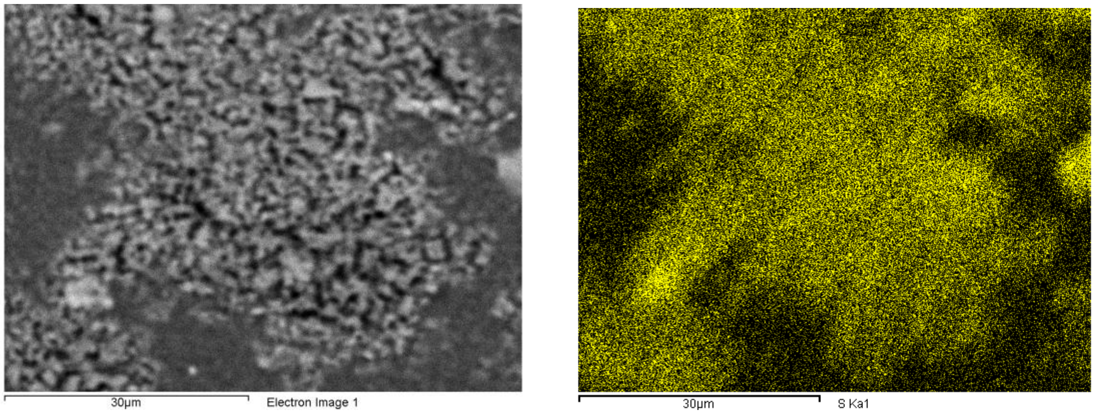

Supplementary Figure 10: SEM and elemental mapping of Sulphur in TTT-COF. SEM image of the TTT-COF (left) and the corresponding EDX map of sulphur (right), where the dark areas indicate the substrate. Due to the instrument-inherent drift, higher resolution of the EDX mapping was not possible.

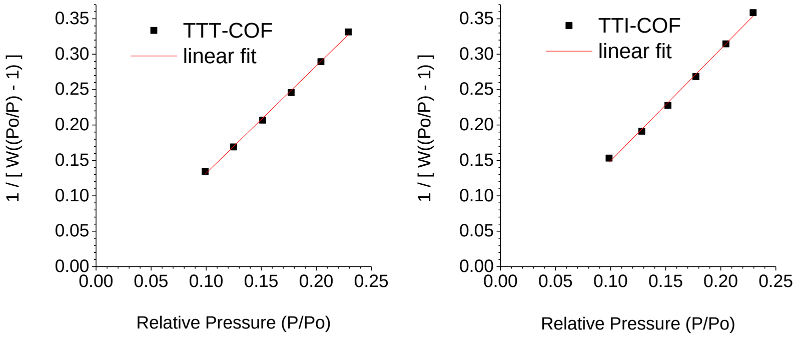

Supplementary Figure 11: BET fit of TTT-COF (left) and TTI-COF (right).

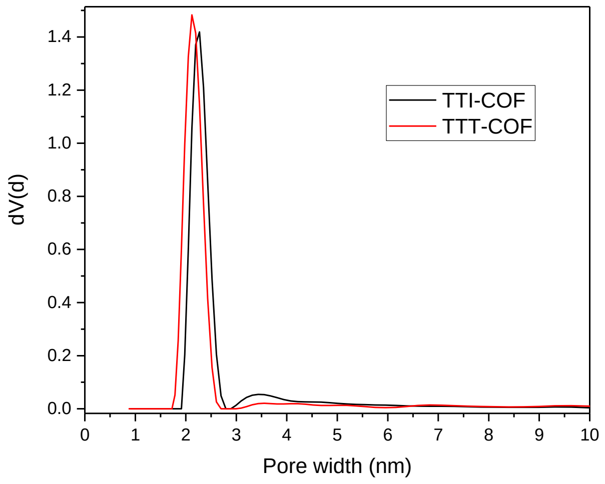

Supplementary Figure 12: Pore size distribution of the TTI-COF and the TTI-COF. PSDs were calculated from Argon isotherms with a QSDFT model based on carbon. The PSD exhibits a small reduction of the average pore size from the TTI-COF to the TTT- COF that matches well with the respective structure models.

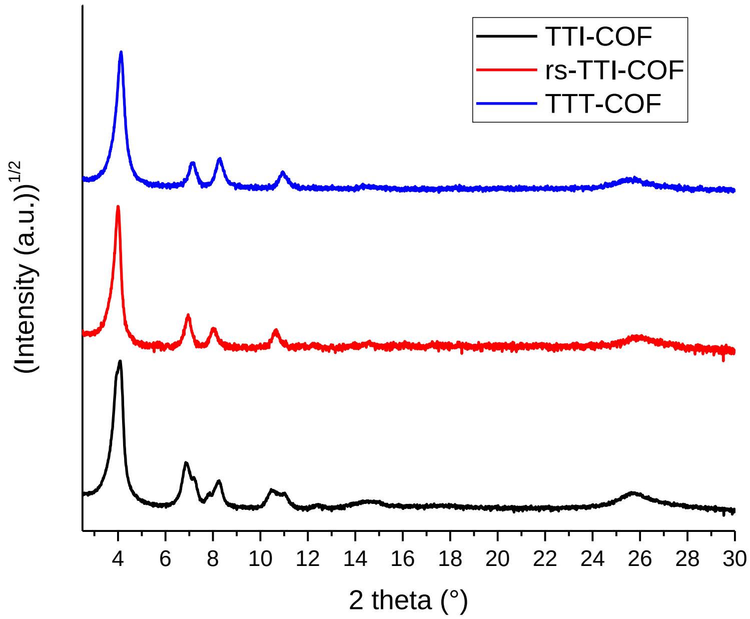

Supplementary Figure 13: Comparison of XRPD pattern in the TT system: randomly stacked (rs) TTI-COF, the TTI-COF (see also ref: [3]) and the TTT-COF.

Supplementary Table 6: Rietveld refinement of the TTI- and TTT-COF.

**Fitted Pattern**	**TTI-COF[3]**	**rs-TTI-COF**	**TTT-COF**
**Space group**	*P*1	$P6_3/m$	$P6_3/m$
**constraints**	*a*=*b* ; *α* = β	-	-
**Rwp (%)**	7.135	11.014	11.091
a **(Å)**	26.060 (8)	25.244 (8)	24.478 (5)
b **(Å)**	26.060 (8)	25.244 (8)	24.478 (5)
c **(Å)**	7.348 (5)	6.905 (7)	7.002 (5)
α **(°)**	80.151 (3)	90	90
**β (°)**	80.151 (3)	90	90
**γ (°)**	120	120	120

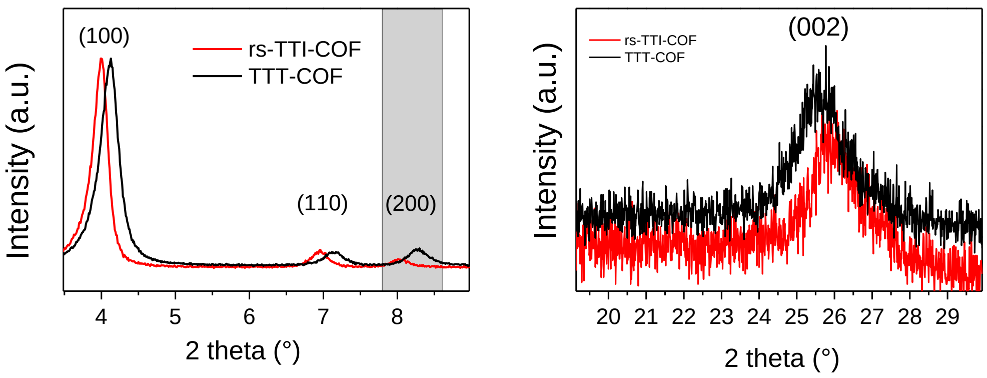

Supplementary Figure 14: Zoomed sections of the XRPD of the TTI and TTT-COF. -These show the shift in the unit cell dimensions (left) and in interlayer distance (right).

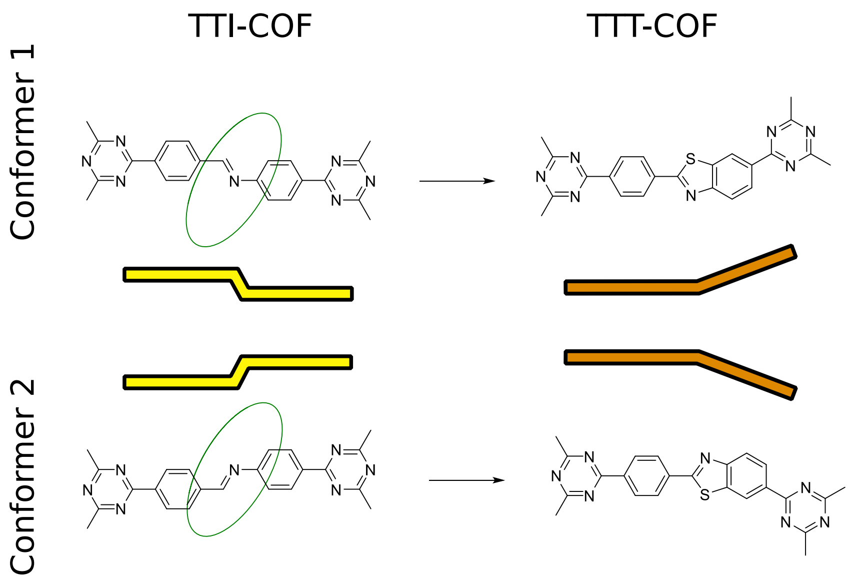

Supplementary Figure 15: Schematic of the conformational isomers. Two conformational isomers in the TTI-COF showing parallel offset (left), with small structural differences between the conformers. However, transformation of these conformers into their respective thiazoles leads to the generation of bending instead of offsets.

Supplementary Table 7 Rietveld analysis of the TTT-COF using different structure models.

**Applied Model**	**imine-model**	**thiazole-model**
**Space group**	$P6_3/m$	$P6_3/m$
**Rwp (%)**	16.237	11.091
a **(Å)**	24.514 (8)	24.478 (5)
b **(Å)**	24.514 (8)	24.478 (5)
c **(Å)**	7.013 (6)	7.002 (5)
α **(°)**	90	90
**β (°)**	90	90
**γ (°)**	120	120

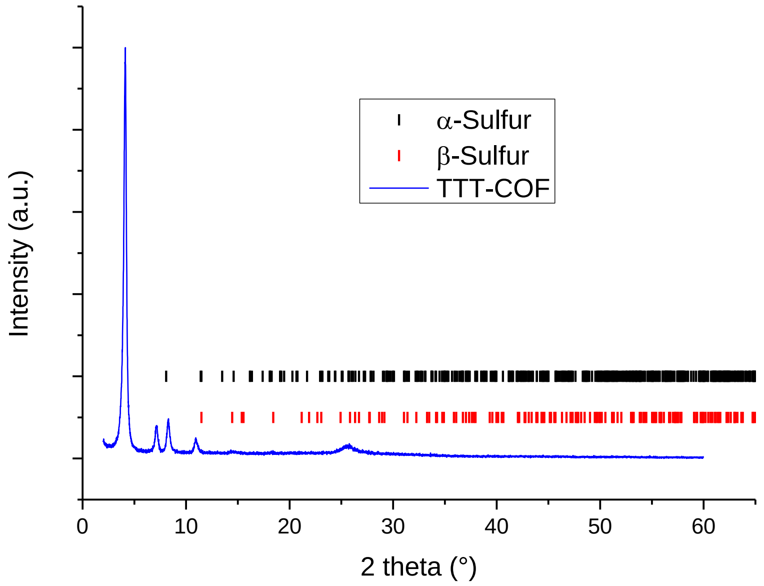

Supplementary Figure 16: Full XRPD of the TTT-COF. The overlay of calculated peak positions of alpha and beta sulphur, shows the absence of sulphur reflections in the measured XRPD.

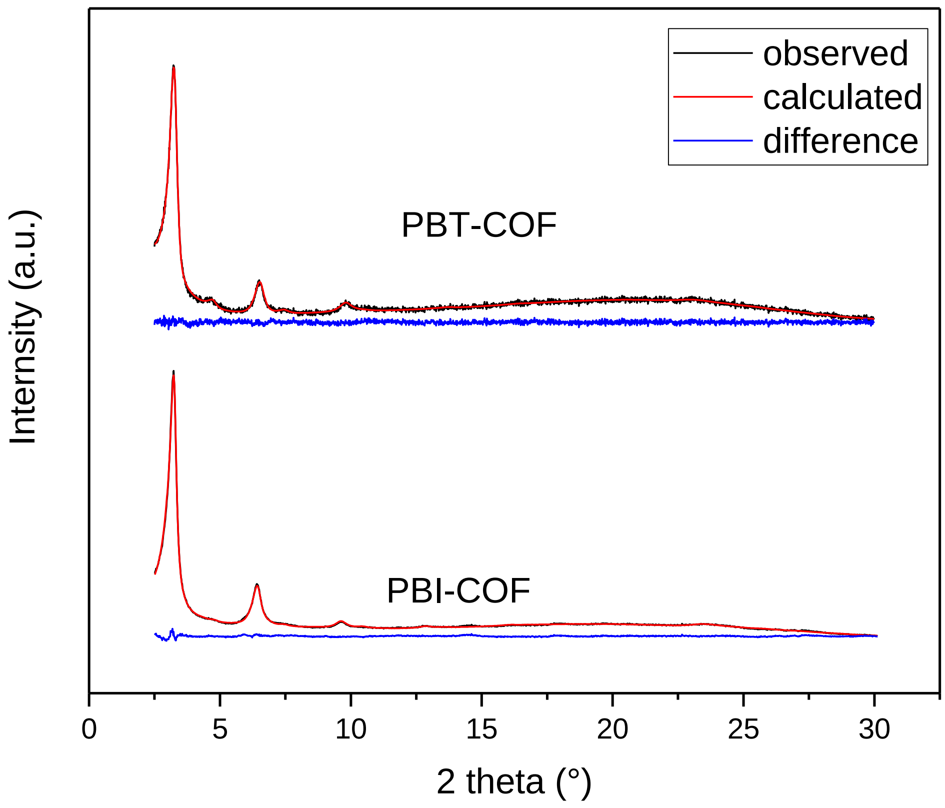

Supplementary Figure 17: XRPD of the PBT- and the PBI-COF. The crystallinity PBI-COF (bottom) was retained after after sulphur incorporation to the PBT-COF (top) and a good match to the Rietveld fit of the respective models.

Supplementary Table 8: Rietveld refinement of the PBI- and PBT-COFs.

**Fitted Pattern**	**PBI-COF**	**PBT-COF**
**Space group**	*P*1	*P*1
**constraints**	*a*=*b* ; *α* = β	*a*=*b* ; α = β
**Rwp (%)**	1.661	3.090
a **(Å)**	28.37 (13)	27.77 (18)
b **(Å)**	28.37 (13)	27.77 (18)
c **(Å)**	3.915 (16)	4.00 (5)
α **(°)**	78.1 (1.3)	76.4 (1.6)
**β (°)**	78.1 (1.3)	76.4 (1.6)
**γ (°)**	82.3 (5)	84.0 (7)

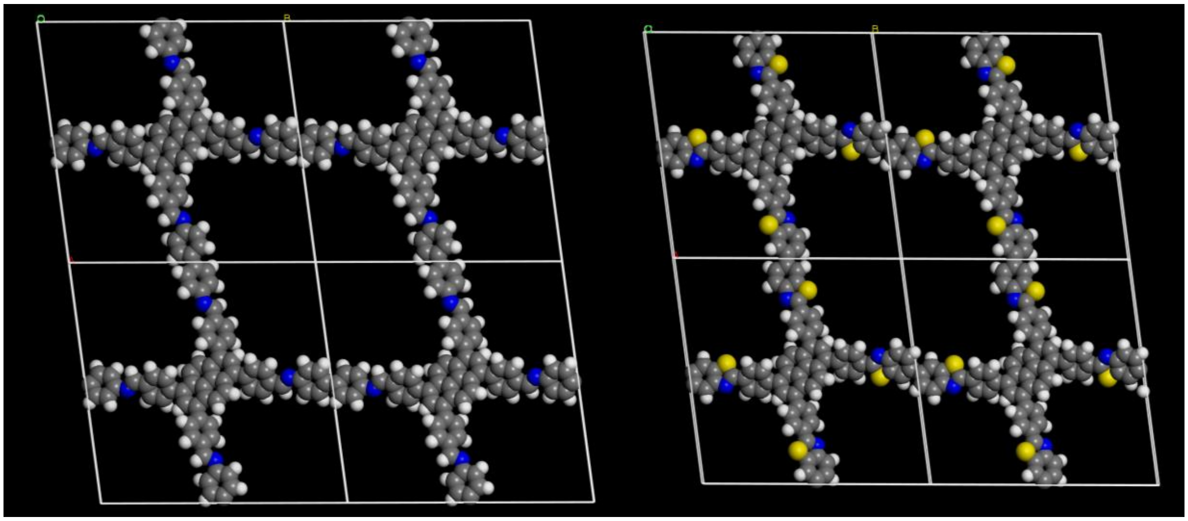

Supplementary Figure 18: Space filing models of the PBI and PBT-COFs. Simulated unit cells of the PBI-COF (left) and the PBT-COF (right).

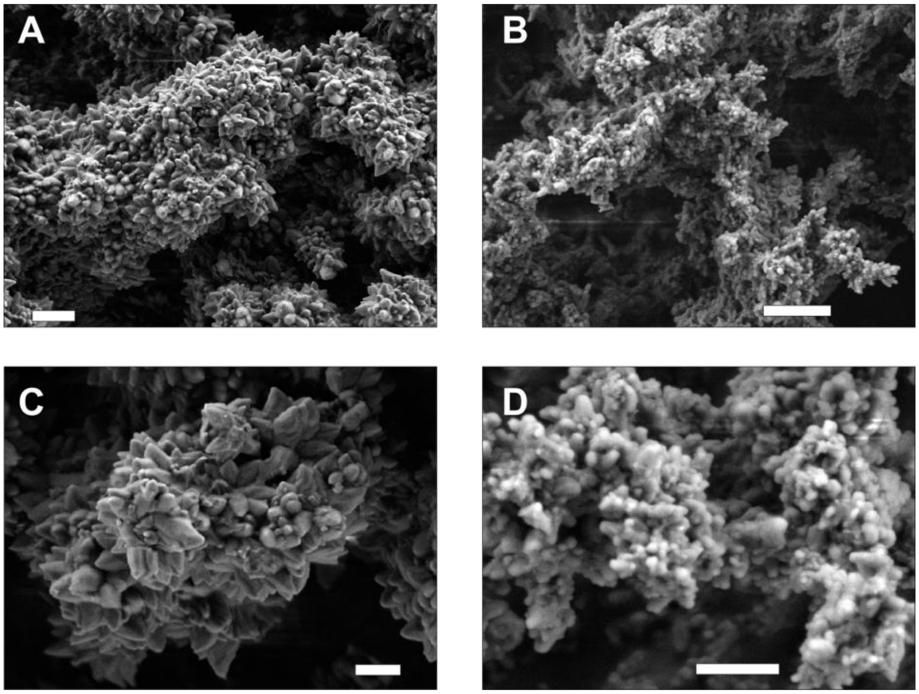

Supplementary Figure 19: SEM images of the investigated COFs. TTI-COF (left, A,C) and the TTT-COF (right, B, D) are shown at different magnifications. Scale bar A,B: 1000 nm; C,D: 500 nm.

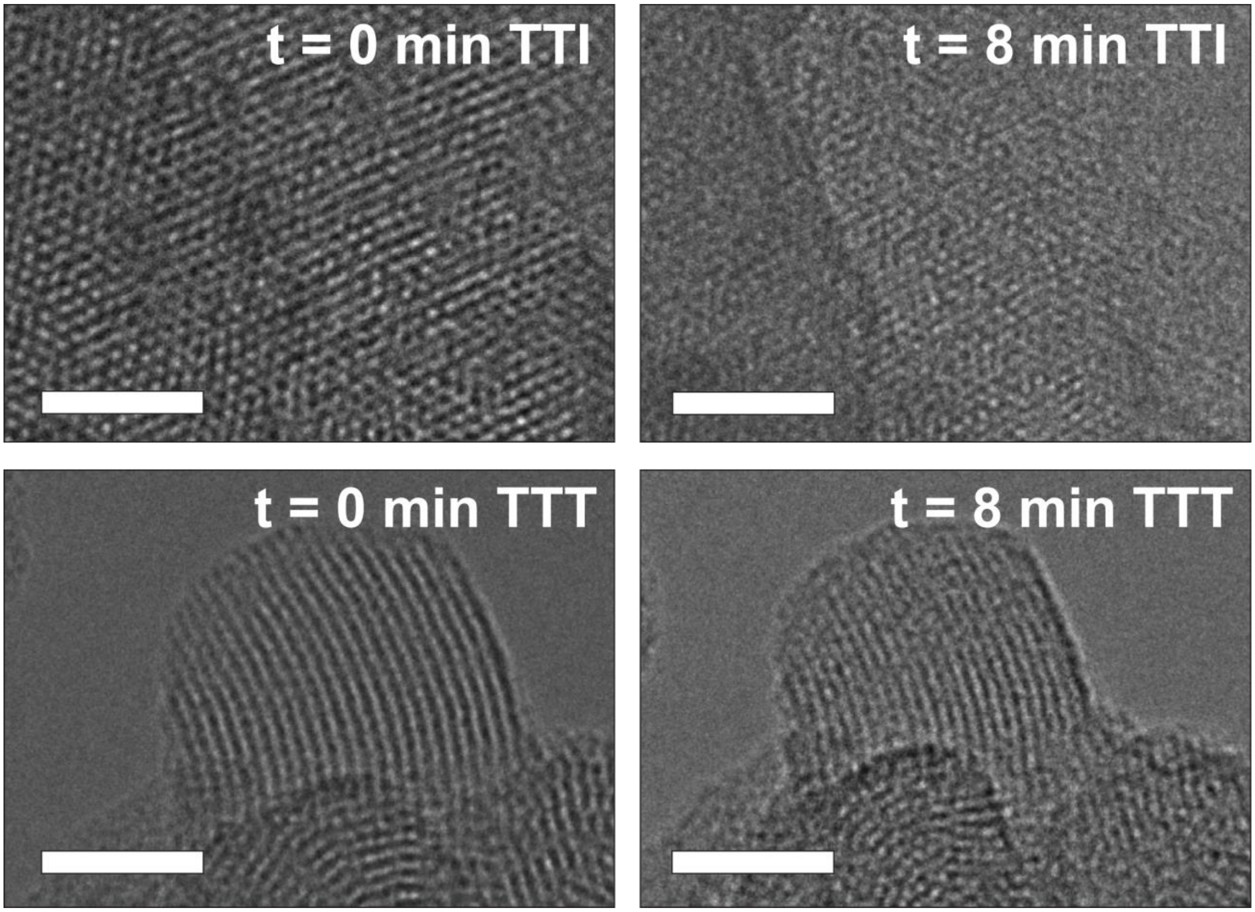

Supplementary Figure 20: Electron beam damage in TTI- and TTT-COF. Different degrees of electron beam damage after 8 minutes of exposure to the electron beam to the TTI-COF (top) and the TTT-COF (bottom) at identical magnifications and electron flux, evident by the reduction of visible lattice fringes. Scale bars: 20 nm.

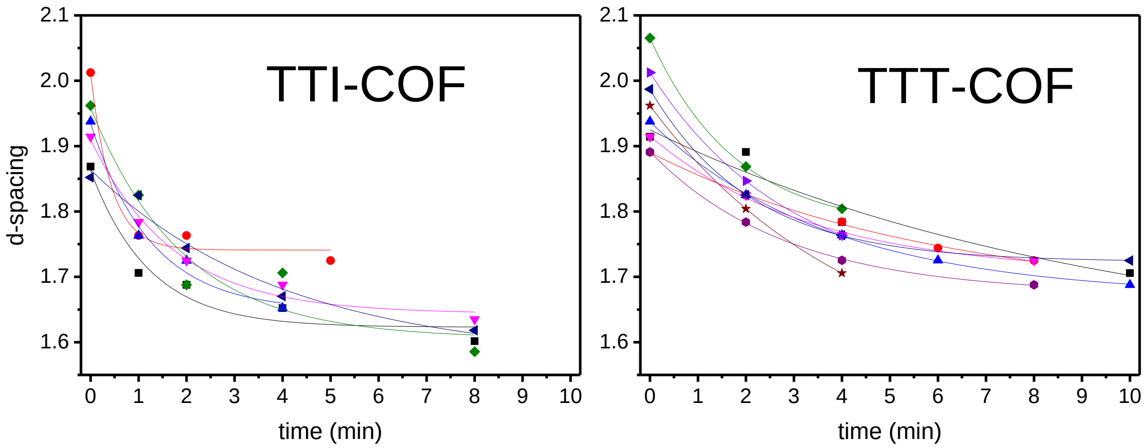

Supplementary Figure 21: Fitting of exponential decays in the fourier transforms of the TEM images The peak positions were extracted from the fourier transforms of the TEM images and were used for the fitting of the half-lives.

Supplementary Table 9: Exemplary fit parameters for the exponential decay in Figure 5 B.

**Model**	ExpDec1	
**Equation**	y = A1*exp(-x/t1) + y0	
**Plot**	**TTI-COF**	**TTT-COF**
**y0**	1.64151 ± 0.01001	1.63748 ± 0.00889
**A1**	0.2582 ± 0.01256	0.26629 ± 0.00923
**t1**	1.87451 ± 0.22461	3.20317 ± 0.26763
**$t_{1/2}$ (half life time)**	1.29931 ± 0.15568	2.22027 ± 0.18550
**Reduced Chi-Sqr**	9.14005E-5	2.24183E-5
**R-Square(COD)**	0.99564	0.99933
**Adj. R-Square**	0.99127	0.99798

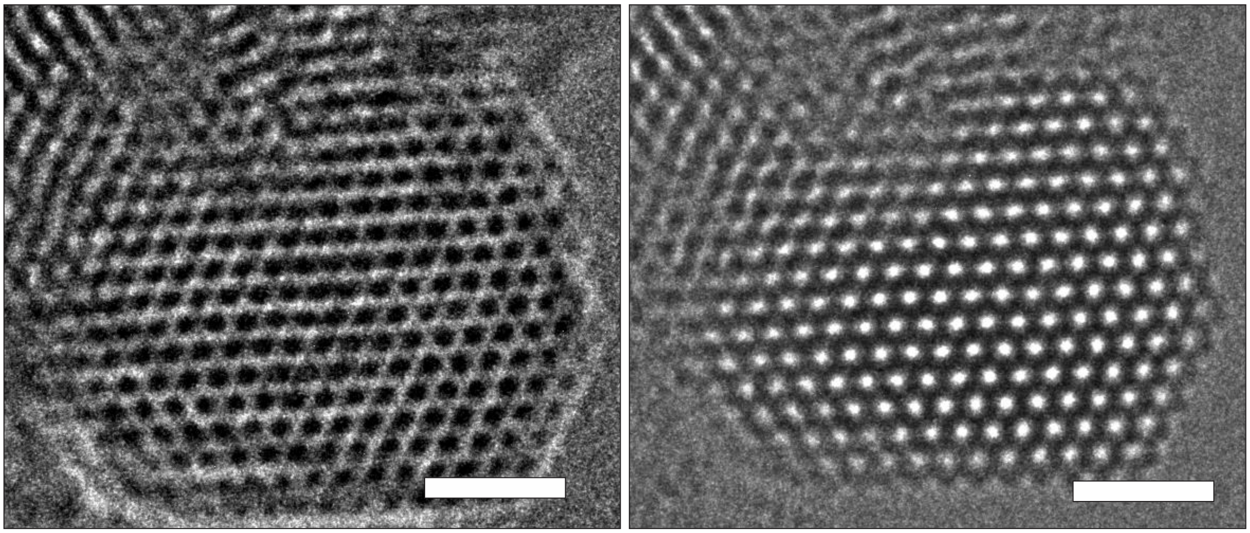

Supplementary Figure 22: Defocus series of a crystallite of the TTT-COF in the TEM along [001]. Scale bar: 10 nm.

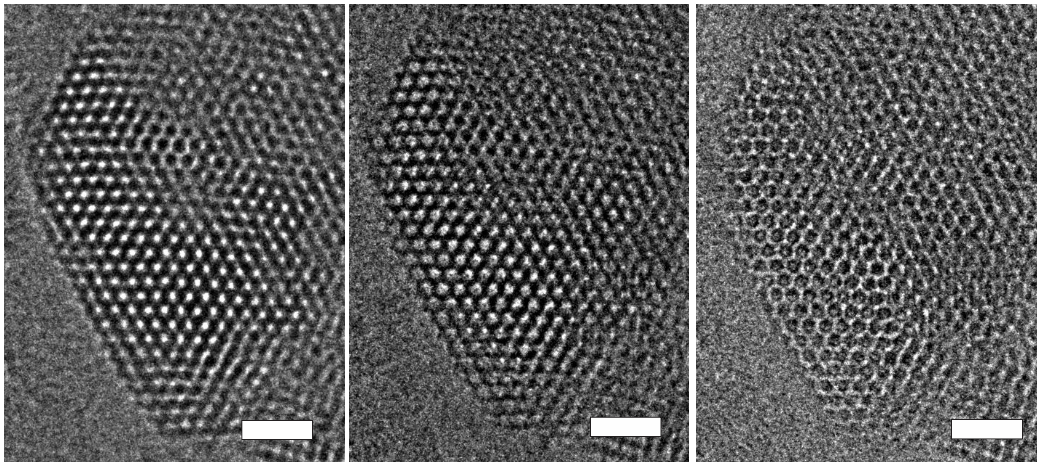

Supplementary Figure 23: Defocus series of a crystallite of the TTT-COF in the TEM along [001]. Scale bar: 10 nm.

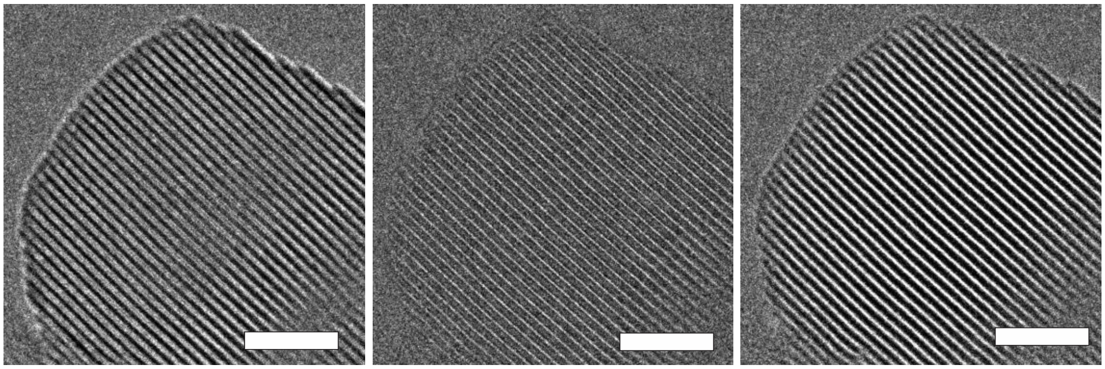

Supplementary Figure 24: Defocus series of the TTT-COF along [100]. Scale bar: 20 nm.

Supplementary Discussion

Molecular nature of edge dislocation in TTT-COF

The molecular nature of the edge dislocation could be described by either a columnar linker vacancy or an out-of-plane growth that resembles a five and a seven membered ring such as spirals (Supplementary Figure 25, Supplementary Figure 26, Figure 6 P-Q). However, the formation of an actual five or a seven membered ring within the COF plane is not possible, since TTI-COF and by extension TTT-COF is composed of two different building blocks that always have to alternate due to their molecular functionalities – the aldehyde building block can only form a bond with the amine and vice versa (Supplementary Figure 26 E A). While true (i.e. in-plane) five and seven-membered rings are not possible in C3-C3 linked COFs, the same does not apply for C3-C2 linked COFs. The latter have less binding and geometry constraints that render the formation of rings with five or seven edges possible. The combination of C3-C3 COFs could thus be used as a strategy for improving crystallinity in 2D COFs by making these in-plane defects less favourable.

In C3+C2 systems the formation of pentagonal or heptagonal rings is possible as the alternation is not between corners but between edge and corner nodes (see Supplementary Figure 25). Of course an uneven

number of building blocks in these structures is also not possible, but the twice as high number of building blocks involved in the ring formation (i.e. building blocks in C2+C3 always have to come "in pairs") results in a higher conformational flexibility that makes these pentagonal and heptagonal rings more likely. However, connecting one layer to the next in C3+C3 systems via a fivefold or a sevenfold screw axis would allow all valences of the linkers to be saturated (Supplementary Figure 26, B, C and D). The introduction of such an edge dislocation into the structure would lead to significant localized strain, caused by the unit cell mismatch as well as the bending strain imposed by the presence of screw axes as they deviate from the ideal angle of 120° in a hexagon. However, such strain and deviation from the ideal structure of the COF matches the observed large reflection broadening that is usually present in COFs. This is in contrast to a model involving a column of vacancies as in Figure 6 Q, Supplementary Figure 26 E, running uniformly along the *c*-direction, where the amount of strain should be much smaller but a large number of linker end groups would remain at the site of the edge dislocation after COF formation. However, it is considered rather unlikely that an in-plane vacancy acts as a "seed" for a column of identical vacancies forming along the out-of-plane direction as sketched in Supplementary Figure 26 E.

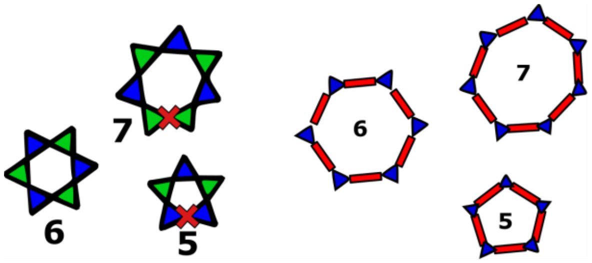

Supplementary Figure 25: Schematic of generating pentagonal and heptagonal rings. Comparison of C3+C3 systems with C3+C2 based systems with regard to their ability to produce pentagonal and heptagonal rings.

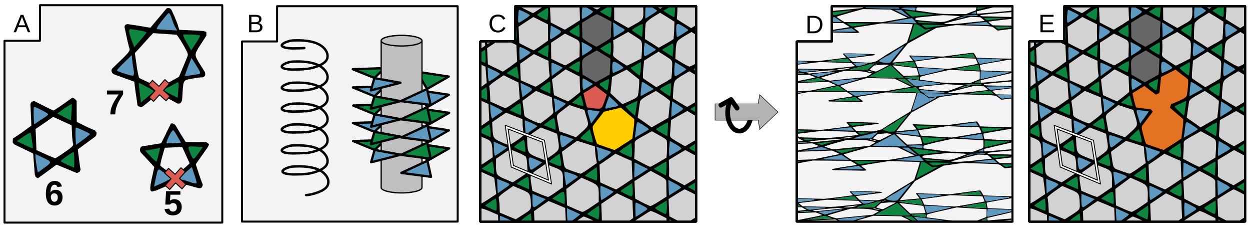

Supplementary Figure 26: Visualizing the formation of screw defects in COFs. A: Schematic of the different kinds of possible (6) and impossible (5,7) in-plane connectivity in a COF composed of two complementary building blocks (green and blue triangles) where alternation between the blue and green triangles enables only even-membered rings. B: Screw axis with an odd number of members per turn, maintaining both in-plane and out-of plane alternation, which look in projection like an odd numbered ring. C, E: View onto the hexagonal plane of the COF with an edge dislocation composed of a vacancy (E) or a screw axis (C). D: Visualization along the COF layers showing the connection of layers by the screw axis, with a greatly exaggerated interlayer distance.

Supplementary References

1 Vyas, V. S. *et al.* Exploiting Noncovalent Interactions in an Imine-Based Covalent Organic Framework for Quercetin Delivery. *Adv. Mater.* **28**, 8749-8754, doi:10.1002/adma.201603006 (2016).

2 Rabbani, M. G., Sekizkardes, A. K., El-Kadri, O. M., Kaafarani, B. R. & El-Kaderi, H. M. Pyrene-directed growth of nanoporous benzimidazole-linked nanofibers and their application to selective CO2 capture and separation. *J. Mater. Chem.* **22**, 25409-25417, doi:10.1039/C2JM34922A (2012).

3 Haase, F. *et al.* Tuning the stacking behaviour of a 2D covalent organic framework through non-covalent interactions. *Mater. Chem. Front.* **1**, 1354-1361, doi:10.1039/c6qm00378h (2017).

4 Leng, W. *et al.* Sophisticated Design of Covalent Organic Frameworks with Controllable Bimetallic Docking for a Cascade Reaction. *Chem. Eur. J.* **22**, 9087-9091, doi:10.1002/chem.201601334 (2016).
